# Interments in the City and Liberties of Philadelphia, from the 3d of July, to the 10th of July

**Published:** 1841-07-17

**Authors:** 


### HEALTH OF THE CITY.

INTERMENTS *in the City and Liberties of Philadelphia, from the 3d of July, to the 10th of July.*

| Diseases. | Adults. | Children. | Diseases. | Adults. | Children. |
|---|---|---|---|---|---|
| Asthama, | 1 | 0 | *Brought forward,* | 35 | 56 |
| Abscess, | 1 | 1 | Inanition, | 0 | 1 |
| Apoplexy, | 3 | 0 | Jaundice, | 1 | 0 |
| Cachexia, | 1 | 0 | Marasmus, | 0 | 6 |
| Cancer of the Womb, | 1 | 0 | Measles, | 0 | 1 |
| Casualties, | 1 | 1 | Mortification of Bowels, | 0 | 1 |
| Croup, | 0 | 2 | Old age, | 4 | 0 |
| Cholera Morbus, | 2 | 0 | Palsy, | 1 | 0 |
| Consumption of the lungs, | 8 | 1 | Softening of brain, | 0 | 1 |
| Concussion of Brain, | 0 | 1 | Small pox, | 0 | 3 |
| Convulsions, | 1 | 8 | Still-born, | 0 | 11 |
| Diarrhœa, | 0 | 10 | Suicide, | 1 | 1 |
| Dropsy, | 1 | 0 | Summer Complaint, | 0 | 19 |
| —— Abdominal, | 1 | 0 | Spitting of Blood, | 2 | 0 |
| —— Head, | 0 | 4 | Tabes Mesenterica | 0 | 1 |
| —— Breast, | 1 | 0 | Unknown, | 1 | 2 |
| Disease of Brain, | 0 | 2 | Total, 148— | 45 | 103 |
| —— Stomach, | 1 | 0 | | | |
| Drowned, | 2 | 1 | | | |
| Dysentery, | 5 | 3 | | | |
| Debility, | 0 | 4 | | | |
| Erysipelas, | 1 | 0 | | | |
| Enlargement of Heart, | 1 | 0 | | | |
| Effusion on Brain, | 0 | 1 | | | |
| Fever, | 0 | 1 | | | |
| Typhus, | 1 | 0 | | | |
| —— Scarlet, | 0 | 2 | | | |
| Inflammation of the Brain, | 1 | 5 | | | |
| —— Bronchi, | 1 | 0 | | | |
| —— Lungs, | 0 | 4 | | | |
| —— Stomach and Bowels, | 0 | 1 | | | |
| —— Bowels, | 1 | 2 | | | |
| —— Breast, | 0 | 1 | | | |
| —— Peritonæum, | 0 | 1 | | | |
| *Carried forward,* | 35 | 56 | | | |

| | |
|---|---|
| Of the above, there were under 1 year, | 59 |
| From 1 to 2 | 22 |
| 2 to 5 | 19 |
| 5 to 10 | 0 |
| 10 to 15 | 2 |
| 15 to 20 | 1 |
| 20 to 30 | 11 |
| 30 to 40 | 10 |
| 40 to 50 | 5 |
| 50 to 60 | 5 |
| 60 to 70 | 6 |
| 70 to 80 | 7 |
| 80 to 90 | 1 |
| 90 to 100 | 0 |
| Total, | 148 |

Of the above there were 9 from the almshouse, 10 people of colour, and 2 from the country, which are included in the total amount.

